# Supplementary material for: Minimal functional domains of the core polarity regulator Dlg
Source: Biol Open. 2022 Jul 25;11(7):bio059408. doi: 10.1242/bio.059408 (PMC9346270; doi:10.1242/bio.059408)
Supplement: Supplementary information [file biolopen-11-059408-s1.pdf]

Biology Open (2022): doi:10.1242/bio.059408: Supplementary information

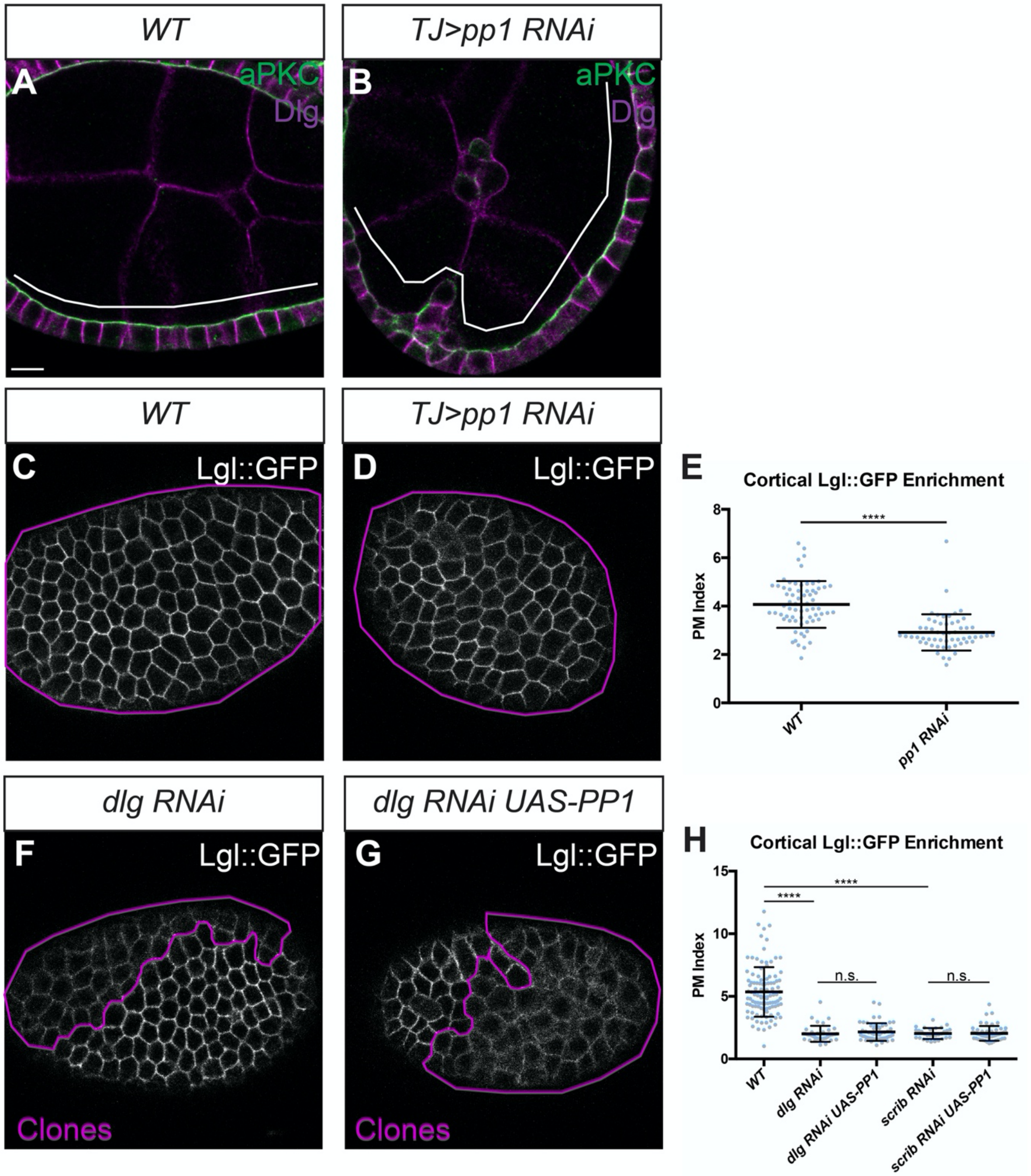

Fig. S1. Lgl localization requires both PP1 and Scrib/Dlg

Compared to WT cells (A), *pp1*-depleted cells (B) exhibit mild polarity loss and occasional multilayering. Compared to WT cells (C), *pp1*-depleted cells display mild loss of cortical Lgl (D, also compare to *dlg*-depletion in F). (E) Quantification of Lgl localization. (F) *dlg*-depleted cells strongly mislocalize cortical Lgl and this is not rescued by overexpression of PP1 (G). (H) Quantification of Lgl localization. Scale bars, 10μm. White lines in (F-G) indicate flip-out GAL4 clones of given genotypes. (E) Two-tailed t-test with Welch's correction. (H) One-way ANOVA with Tukey's multiple comparisons test. Error bars indicate S.D. PM Index=cortical/cytoplasmic intensity. Data points are individual cell measurements. n.s. (not significant) $P > 0.05$, ****$P < 0.0001$.

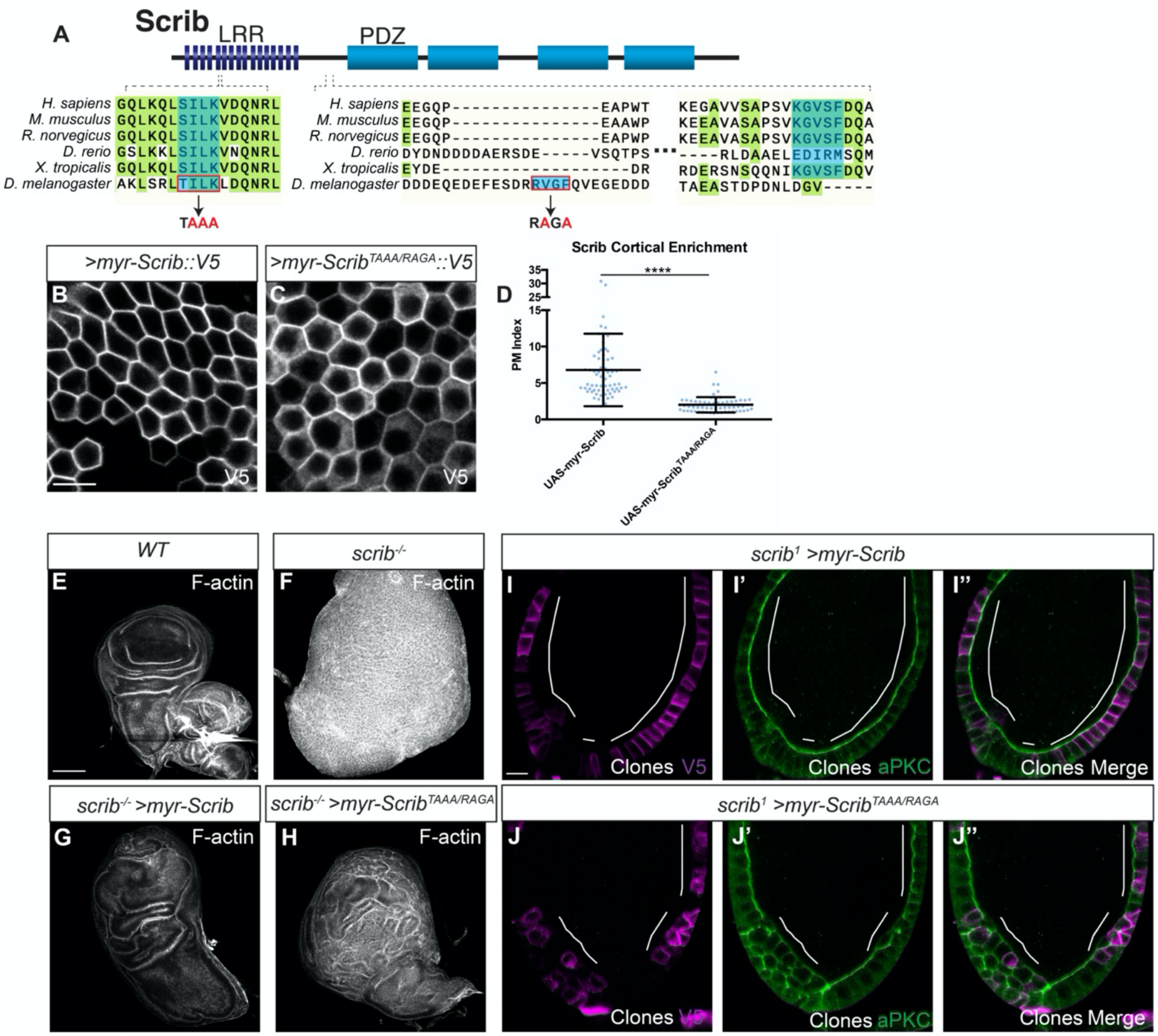

Fig. S2. Scrib PP1-binding consensus motifs are partially required for function

(A) Cartoon showing the Scrib protein domain composition and location of the SILK and RVxF motifs. Below: alignment showing conservation of the SILK motif and RVxF motifs. Note that in vertebrates (right), the RVxF motif is located slightly C-terminal to its position in insects (left). Red boxes indicate residues mutated in myr-$^{\text{ScribTAAA/RAGA}}$ construct. Compared to WT myr-Scrib (B), myr-Scrib$^{\text{TAAA/RAGA}}$ (C) localizes less well to the cell cortex but is still enriched at the basolateral membrane, quantified in (D). Both constructs contain V5 epitope tags, used for detection. (E-H) Compared to WT wing discs (E), *scrib* mutant wing discs overgrow and form tumors (F). Overexpression of myr-Scrib largely rescues these phenotypes (G), while expression of myr-Scrib$^{\text{TAAA/RAGA}}$ only partially rescues the *scrib* mutant phenotype (H). (I-J) Compared to myr-Scrib (I), myr-Scrib$^{\text{TAAA/RAGA}}$ (J) provides less efficient rescue of *scrib* mutant: myr-Scrib shows complete restoration of the monolayered epithelium in 78.6% (n=14) of follicles, compared to complete restoration in 36.8% (n=19) of myr-Scrib$^{\text{TAAA/RAGA}}$ -rescued follicles. Scale bars, 10μm, except E-H, 100μm. White lines in (I-J) indicate MARCM clones of given genotypes, (B-C) are flip-out GAL4 clones. (D) Two-tailed t-test with Welch's correction. Error bars indicate S.D. PM Index=cortical/cytoplasmic intensity. Data points are individual cell measurements. ****P < 0.0001.

Biology Open • Supplementary information

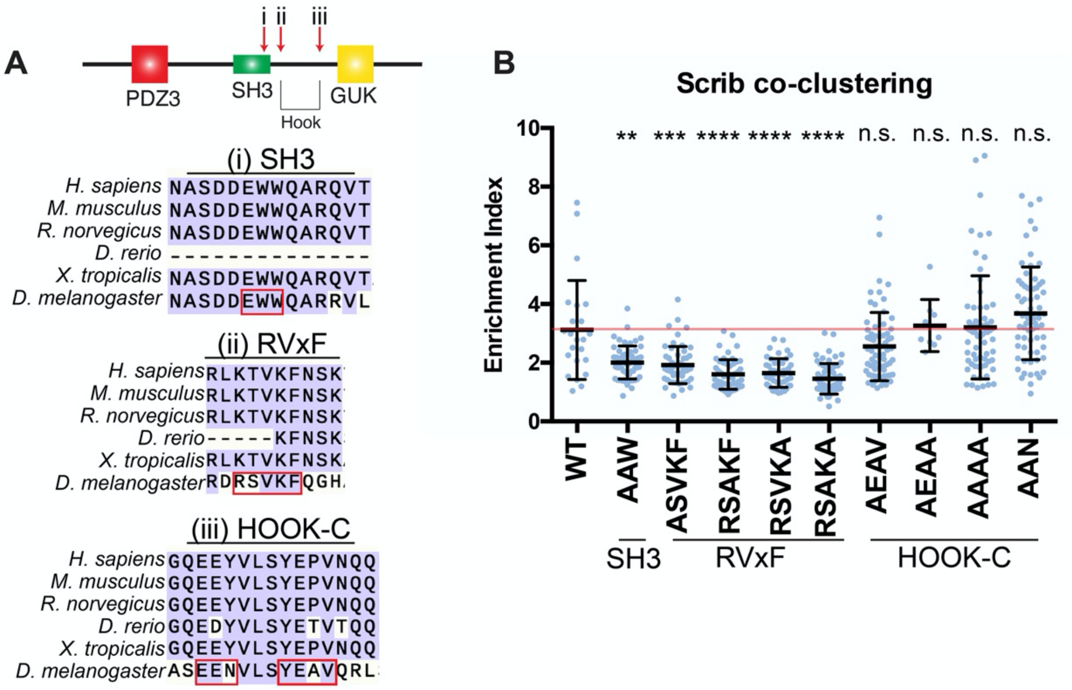

Fig. S3. In depth examination of Dlg SH3 and HOOK domains

(A) Schematic of the Dlg domains used in the $\text{Ed-Dlg}^{\text{PDZ3-SH3-HOOK-GUK}}$ construct, with sequence alignments showing conservation of the SH3 and HOOK domain sequences chosen for study. Motifs targeted for mutation are indicated by red outlines. Arrows in cartoon indicate relative locations of targeted sequences in the protein. (B) Quantification of Scrib recruitment to the polarity site in S2 induced polarity assay. Compared to the WT $\text{Ed-Dlg}^{\text{PDZ3-SH3-HOOK-GUK}}$ construct, which does recruit Scrib, the SH3 mutant AAW construct has reduced ability to recruit Scrib. Similarly, all four constructs targeting single residues of the RVxF motif show equally impaired ability to recruit Scrib. However, the four constructs targeting conserved residues in the C-terminal HOOK domain do not impair Scrib recruitment. Red line indicates the average for the WT construct control. One-way ANOVA with Tukey's multiple comparisons test. Error bars indicate S.D. Data points are individual cell clusters. Statistical tests are comparisons to the $\text{Ed-Dlg}^{\text{PDZ3-SH3-HOOK-GUK}}$ control construct. Enrichment index = contact site/non-contact site intensity. n.s. (not significant) $P > 0.05$, $^{**}P < 0.01$, $^{***}P < 0.001$, $^{****}P < 0.0001$.

Biology Open • Supplementary information

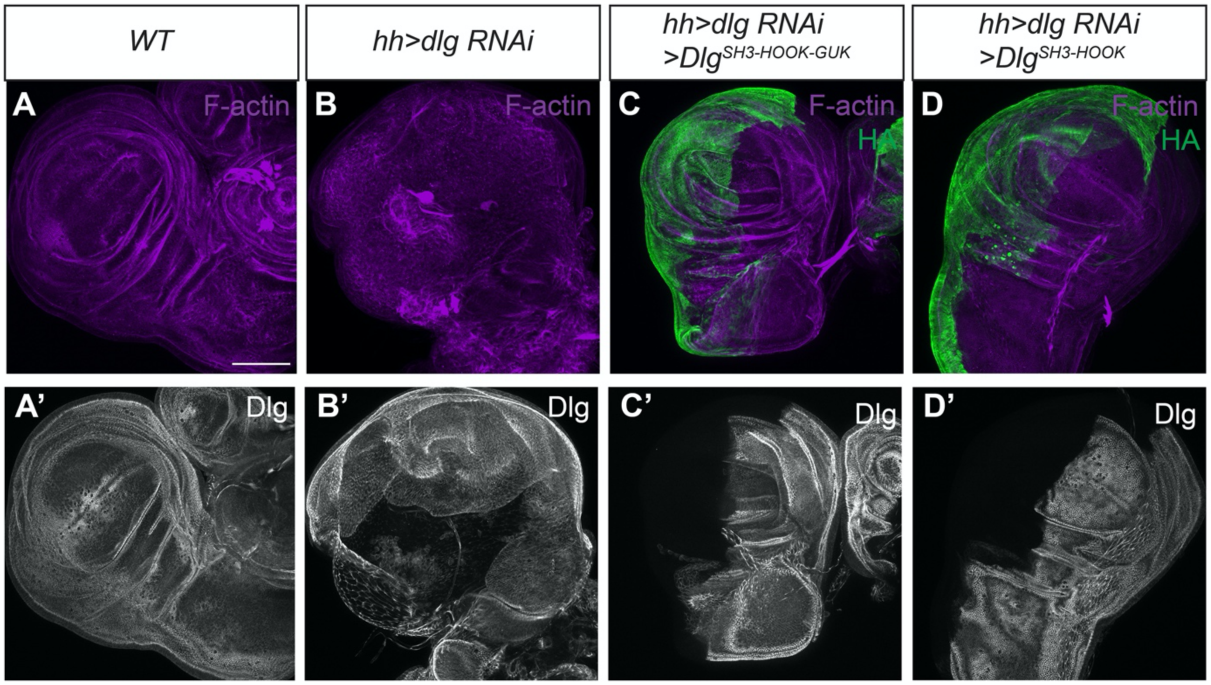

Fig. S4. Validation of RNAi rescue approach for minimal Dlg constructs

(A-D) Knocking down *dlg* in the posterior half of the wing disc using an RNAi construct targeting PDZ2-encoding sequences (B) causes polarity loss and disrupted epithelial architecture. These phenotypes are fully rescued by co-expression of Dlg$^{SH3\text{-}HOOK\text{-}GUK}$ (C) and Dlg$^{SH3\text{-}HOOK}$ (D), and neither constructs is targeted by the RNAi reagent used to deplete endogenous Dlg (C’, D’). Scale bars, 100µm.

Biology Open • Supplementary information

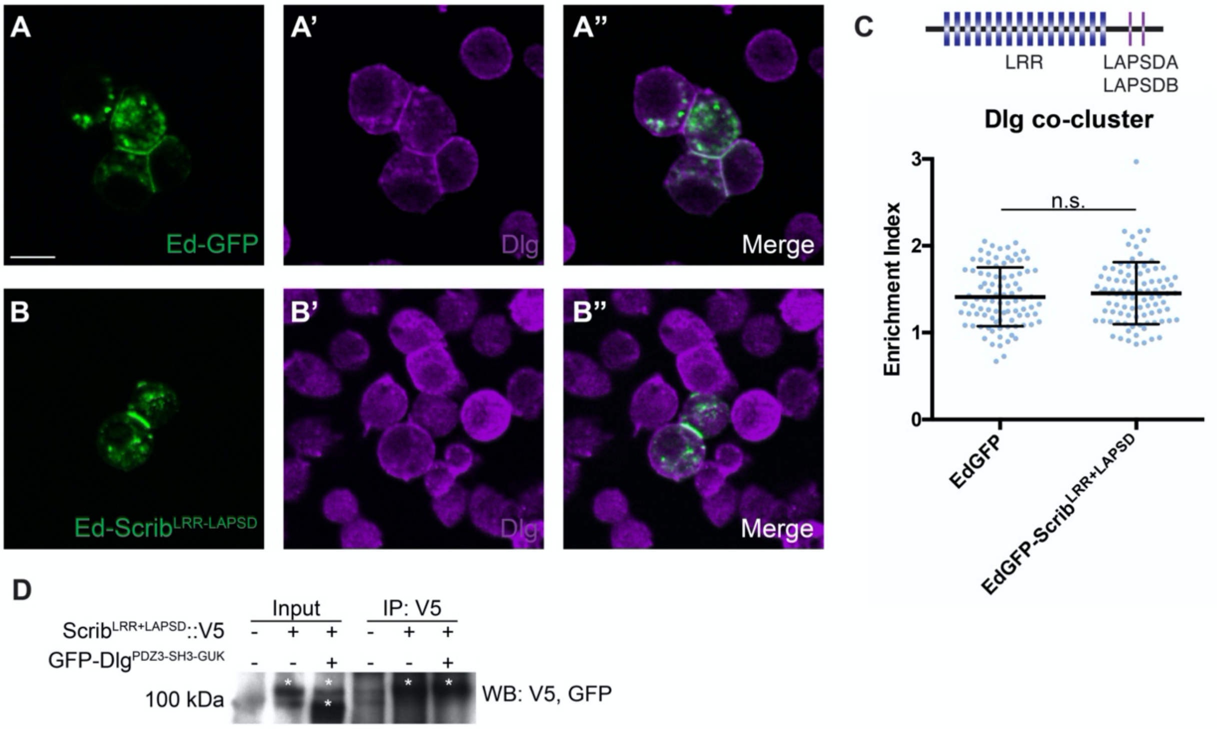

Fig. S5. Using S2 cell induced polarity to study Scrib-Dlg interaction

(A) Ed-GFP expression in S2 cells allows induction of a polarity domain where cells adhere. (B) Fusing the Scrib LRR+LAPSD domains to Ed creates a domain of polarized Scrib. (C) Schematic of the Scrib domains used in the Ed-Scrib$^{LRR+LAPSD}$ construct. Quantification of Dlg enrichment shows that Scrib cannot recruit Dlg to the polarity site. (D) Co-IP assay of Scrib$^{LRR+LAPSD}$ and Dlg$^{PDZ3\text{-}SH3\text{-}HOOK\text{-}GUK}$ from S2 cells fails to detect interaction between these proteins. Scale bars, 10µm. (C) Two-tailed t-test with Welch's correction. Error bars indicate S.D. Data points are individual cell clusters. Enrichment index = contact site/non-contact site intensity. n.s. (not significant).

Biology Open • Supplementary information

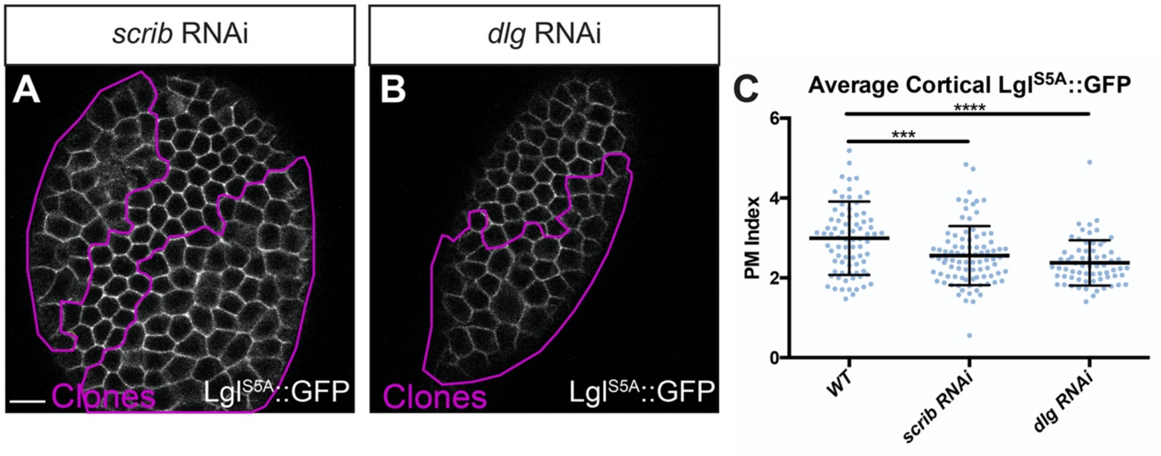

Fig. S6. Scrib and Dlg protection of Lgl is partly independent of phosphorylation
(A-B) Compared to its localization in WT cells, non-phosphorylatable Lgl^{S5A} exhibits a slight, but significant, reduction in cortical levels in *scrib* RNAi (A) and *dlg* RNAi expressing cells (B). (C) Quantification of LglS5A::GFP levels. *scrib* or *dlg* RNAi both significantly reduce Lgl^{S5A} cortical levels compared to WT cells. Scale bars, 10μm. White lines indicate flip-out GAL4 clones of given genotypes. PM Index=cortical/cytoplasmic intensity. Data points represent individual cell measurements. Error bars represent S.D. (C) One-way ANOVA with Dunnett's multiple comparisons test. ***P < 0.001, ****P < 0.0001.

Biology Open • Supplementary information

Table S1. Key Resources

Reagent	Reference and Source
***Drosophila* stocks**	
UAS-Dlg^{WT}::HA	(Sharp et al., 2021)
UAS-Dlg^{ASAKA}::HA	This study
UAS-Dlg$^{SH3\text{-}HOOK}$::HA	This study
UAS-Dlg$^{SH3\text{-}HOOK\text{-}GUK}$::HA	This study
UAS-myr-Scrib::V5	(Khoury and Bilder, 2020)
UAS-myr-Scrib$^{TAAA/RAGA}$::V5	This study
dlg^{m52}	(Perrimon, 1988)
dlg^{m30}	(Woods and Bryant, 1989)
dlg$^{40.2}$	(Mendoza-Topaz et al., 2008)
scrib^{1}	(Bilder and Perrimon, 2000)
scrib^{2}	(Zeitler et al., 2004)
UAS-dlg RNAi HMS01954	Bloomington Drosophila Stock Center (BDSC): 39035
UAS-scrib RNAi HMS01993	BDSC: 39073
UAS-Pp1-87B RNAi HMS00409	BDSC: 32414
UAS-Pp1-87B::HA	BSDC: 24098
UASp-Sds22::GFP	BDSC: 65851
UASp-Scrib::GFP	(Zeitler et al., 2004)
UAS-EGFP::Dlg	(Koh et al., 1999)
UAS-Lgl::GFP	(Wirtz-Peitz et al., 2008)
UAS-Lgl^{KAFA}::GFP	(Moreira et al., 2019), Generously provided by E. Morais de Sá.
Lgl::GFP	(Dong et al., 2015), Generously provided by Y. Hong.
Lgl^{S5A}::GFP	(Dong et al., 2015), Generously provided by Y. Hong
act>y+>GAL4 UAS-his::RFP	BDSC: 30558
tub-GAL80 FRT19A; act-GAL4 UAS-GFP	(Lee and Luo, 1999), BDSC: 42726, 5134
tj-GAL4	Kyoto Stock Center: 104055
hh-GAL4	(Tanimoto et al., 2000)
D174-GAL4	(Sharp et al., 2021)
**Plasmids**	
pMT-Ed-GFP	(Johnston et al., 2009), Generously provided by C. Johnston.
pMT-Ed-GFP-Dlg$^{PDZ3\text{-}SH3\text{-}HOOK\text{-}GUK}$	(Garcia et al., 2014), Generously provided by C. Johnston.
pMT-Ed-GFP-Dlg$^{SH3\text{-}GUK,\Delta HOOK}$	Generously provided by C. Johnston.
pMT-Ed-GFP-Dlg$^{SH3\text{-}GUK}$	This study
pMT-Ed-GFP-Dlg$^{PDZ3\text{-}SH3\text{-}HOOK}$	This study
pMT-Ed-GFP-Dlg^{SH3}	This study
pMT-Ed-GFP-Dlg^{HOOK}	This study
pMT-Ed-GFP-Dlg$^{SH3\text{-}HOOK}$	This study
pMT-Ed-GFP-Dlg$^{PDZ3\text{-}SH3\text{-}HOOK\text{-}GUK[m30]}$	This study
pMT-Ed-GFP-Dlg$^{PDZ3\text{-}SH3\text{-}HOOK\text{-}GUK[ASAKA]}$	This study
pMT-Ed-GFP-Dlg$^{PDZ3\text{-}SH3\text{-}HOOK\text{-}GUK[ASVKF]}$	This study

Biology Open • Supplementary information

pMT-Ed-GFP-Dlg$^{PDZ3-SH3-HOOK-GUK[RSAKF]}$	This study
pMT-Ed-GFP-Dlg$^{PDZ3-SH3-HOOK-GUK[RSVKA]}$	This study
pMT-Ed-GFP-Dlg$^{PDZ3-SH3-HOOK-GUK[RSAKA]}$	This study
pMT-Ed-GFP-Dlg$^{PDZ3-SH3-HOOK-GUK[AEAV]}$	This study
pMT-Ed-GFP-Dlg$^{PDZ3-SH3-HOOK-GUK[AEAA]}$	This study
pMT-Ed-GFP-Dlg$^{PDZ3-SH3-HOOK-GUK[AAAA]}$	This study
pMT-Ed-GFP-Dlg$^{PDZ3-SH3-HOOK-GUK[AAN]}$	This study
pMT-Ed-GFP-Dlg$^{PDZ3-SH3-HOOK-GUK[AAW]}$	This study
pMT-Ed-GFP-Scrib$^{LRR+LAPSD}$	This study
pMT-Scrib$^{LRR+LAPSD}$::V5	This study
pMT-GFP::Dlg$^{PDZ3-SH3-HOOK-GUK}$	This study
**Antibodies**	
Mouse anti-Dlg (1:100 IHC)	Developmental Studies Hybridoma Bank (DSHB): 4F3
Rabbit anti-aPKC (1:200 IHC)	Santa Cruz Biotech: sc-216
Guinea pig anti-Scrib (1:500 IHC)	(Bilder and Perrimon, 2000)
Rabbit anti-HA tag C29F4 (1:500 IHC, 1:10,000 WB, 1:200 IP)	Cell Signaling Technologies (CST): #3724
Mouse anti-HA tag 6E2 (1:10,000 WB)	CST: #2367
Mouse anti-HA tag 2-2.2.14 (1:200 IP)	Invitrogen: 26183
Rabbit anti-GFP (1:5000 WB)	Origene: TP-401
Mouse anti-GFP JL-8 (1:10,000 WB)	Clontech: 632380
Mouse anti-V5 (1:500 IHC, 1:200 IP, 1:5000 WB)	Invitrogen: R960-25
**Primers**	
myrScrib_silk_F	tgaatagggaattggggtaccatgggtaactgcctcaccac
myrScrib_Silk_R	tctgatccaacgcggcagcagtcagtct
myrScrib_rvxf_F	tgctgccgcgttggatcagaatcgattgcagcggttgaacgatac
myrScrib_Rvxf_R	cctccacttgggcgccagcggcgcgatc
myrScrib_AgeI_F	cgctggcgcccaagtggagggcgaagatg
myrScrib_AgeI_R	ggtacgacggggagcgggcaccggttgacccgtggaactgtctatc
Nterm_Dlg_F	actctgaatagggaattggctcgagcaaaATGACAACGAGGAAAAAGAAGCGC
Nterm-Dlg-RVxF	tggGCcttaGcgctgGCgtccctagctcgcattttgcg
Dlg-RVxF-Cterm	gacGCCAGCgCtaagGCccagggacatgcggcag
Dlg_Cterm_R	ggttccttcacaaagatcctctagaatcTTAAGCGTAGTCTGGGAC
Dlg-SH3_for	Actctgaatagggaattggctcgagcaaaatgcagtaccgcccagaggag

Biology Open • Supplementary information

Dlg-SH3-HOOK_rev	Ccgactgggagtagttgatggacaaacgctgtac
Dlg-Cterm_for	catcaactactcccagtcgggaccaacc
Ed-PDZ3-SH3-HOOK-GUK m30 F	TGTGCGCGCCCCGTTTGACTACG
Ed-PDZ3-SH3-HOOK-GUK m30 R	TACAGCGATCGCTTTTGCG
Ed-PDZ3-SH3-HOOK-GUK ASAKA F	TAAGGCCCAGGGACATGCGGCAGCT
Ed-PDZ3-SH3-HOOK-GUK ASAKA R	GCGCTGGCGTCCCTAGCTCGCATTTTGC
Ed-PDZ3-SH3-HOOK-GUK delPDZ3 F	GAGGAGTACAATCGCTTCG
Ed- PDZ3-SH3-HOOK-GUK delPDZ3 R	GCGCGGTTCTCTGGTTAT
Ed- PDZ3-SH3-HOOK-GUK delGUK F	TCCCAGTCGGGACCAACC
Ed- PDZ3-SH3-HOOK-GUK delGUK R	GTAGTTGATGGACAAACGCTGTAC
Ed- PDZ3-SH3-HOOK-GUK deltaSH3_F	CGAGCTAGGGACCGCAGC
Ed- PDZ3-SH3-HOOK-GUK deltaSH3_R	TTGCGTGGTGCGCAGCAG
Ed- PDZ3-SH3-HOOK-GUK ASVKF_F	TAAGTTCCAGGGACATGCGGCAGCT
Ed- PDZ3-SH3-HOOK-GUK ASVKF_R	ACGCTGGCGTCCCTAGCTCGCATTTTGC
Ed- PDZ3-SH3-HOOK-GUK RSAKF_F	TAAGTTCCAGGGACATGCGGCAGCT
Ed- PDZ3-SH3-HOOK-GUK RSAKF_R	GCGCTGCGGTCCCTAGCTCGCATTTTGC
Ed- PDZ3-SH3-HOOK-GUK RSVKA_F	TAAGGCCCAGGGACATGCGGCAGCT
Ed- PDZ3-SH3-HOOK-GUK RSVKA_R	ACGCTGCGGTCCCTAGCTCGCATTTTGC
Ed- PDZ3-SH3-HOOK-GUK RSAKA_F	TAAGGCCCAGGGACATGCGGCAGCT
Ed- PDZ3-SH3-HOOK-GUK RSAKA_R	GCGCTGCGGTCCCTAGCTCGCATTTTGC
Ed- PDZ3-SH3-HOOK-GUK AAW661/2_F	GTGGCAGGCACGACGAGTTCTC
Ed- PDZ3-SH3-HOOK-GUK AAW661/2_R	GCTGCATCGTCGGAGGCATTGGT
Ed- PDZ3-SH3-HOOK-GUK EEN768_F	GAACGTGTTGTCCTACGAGGCC
Ed- PDZ3-SH3-HOOK-GUK EEN768_R	GCCGCGGAAGCTCCTTCAGCATTG
Ed- PDZ3-SH3-HOOK-GUK AEAV_F	CGTGTTGTCCGCAGAGGCCGTACAGC
Ed- PDZ3-SH3-HOOK-GUK AEAV_R	TTCTCCTCGGAAGCTCCT

Biology Open • Supplementary information

Ed- PDZ3-SH3-HOOK-GUK AEAA_F	GCCGCACAGCGTTTGTCCATCAAC
Ed- PDZ3-SH3-HOOK-GUK AEAA_R	CTCTGCGGACAACACGTTCTCCTC
Ed- PDZ3-SH3-HOOK-GUK AAAA_F	GCCGCACAGCGTTTGTCCATCAAC
Ed- PDZ3-SH3-HOOK-GUK AAAA_R	CGCTGCGGACAACACGTTCTCCTC
Ed_scrib_Gibson_F	gcatggacgagctgtacaagctatgttcaagtgcattcccatcttc
Ed_scrib_Gibson_R	ccttcgaagggccctctagagtcggtgctagcctctgc
GFP_fwd	ctactagtccagtgtggtggatggtgagcaagggcgag
GFP_rev	tgctgacagcggatctcttgtacagctcgtc
PDZ3-SH3-HOOK-GUK_fwd (for cytosolic Dlg)	caagagatccgctgtcagcaccgaggatataac
PDZ3-SH3-HOOK-GUK_rev (for cytosolic Dlg)	aatggtgatggtgatgatgatcatagagattccttggaaggtac
scrib_fwd (for cytosolic Scrib)	ctactagtccagtgtggtggatgttcaagtgcattcccatcttcaag
scrib_rev (for cytosolic Dlg)	cttcgaagggccctctagacgcgtcggtgctagcctct

Table S2. Scrib and Dlg transgenic constructs

**Construct**	**Description**
Dlg^{ASAKA}	RSVKF 675-679 to ASAKA
DlgSH3-HOOK	Fragment encompassing aa564-784+961-975
DlgSH3-HOOK-GUK	Fragment encompassing aa564-975
myr-$Scrib^{TAAA/RAGA}$	TILK 286-289 to TAAA and RVGF 621-624 to RAGA
pMT-Ed-GFP-$Dlg^{PDZ3\text{-}SH3\text{-}HOOK\text{-}GUK}$	Fragment encompassing aa473-975
pMT-Ed-GFP-$Dlg^{SH3\text{-}GUK,\Delta HOOK}$	Fragment encompassing aa597-678+771-975
pMT-Ed-GFP-$Dlg^{SH3\text{-}GUK}$	Fragment encompassing aa568-975
pMT-Ed-GFP-$Dlg^{PDZ3\text{-}SH3\text{-}HOOK}$	Fragment encompassing aa473-784+961-975
pMT-Ed-GFP-Dlg^{SH3}	Fragment encompassing aa597-678+766-783+961-975
pMT-Ed-GFP-Dlg^{HOOK}	Fragment encompassing aa473-485+671-784+961-975
pMT-Ed-GFP-$Dlg^{SH3\text{-}HOOK}$	Fragment encompassing aa473-485+564-784+961-975
pMT-Ed-GFP-$Dlg^{PDZ3\text{-}SH3\text{-}HOOK\text{-}GUK[m30]}$	L608→P
pMT-Ed-GFP-$Dlg^{PDZ3\text{-}SH3\text{-}HOOK\text{-}GUK[ASAKA]}$	RSVKF 675-679 to ASAKA
pMT-Ed-GFP-$Dlg^{PDZ3\text{-}SH3\text{-}HOOK\text{-}GUK[ASVKF]}$	RSVKF 675-679 to ASVKF
pMT-Ed-GFP-$Dlg^{PDZ3\text{-}SH3\text{-}HOOK\text{-}GUK[RSAKF]}$	RSVKF 675-679 to RSAKF
pMT-Ed-GFP-$Dlg^{PDZ3\text{-}SH3\text{-}HOOK\text{-}GUK[RSVKA]}$	RSVKF 675-679 to RSVKA
pMT-Ed-GFP-$Dlg^{PDZ3\text{-}SH3\text{-}HOOK\text{-}GUK[RSAKA]}$	RSVKF 675-679 to RSAKA
pMT-Ed-GFP-$Dlg^{PDZ3\text{-}SH3\text{-}HOOK\text{-}GUK[AEAV]}$	YEAV774-777 to AEAV
pMT-Ed-GFP-$Dlg^{PDZ3\text{-}SH3\text{-}HOOK\text{-}GUK[AEAA]}$	YEAV774-777 to AEAA
pMT-Ed-GFP-$Dlg^{PDZ3\text{-}SH3\text{-}HOOK\text{-}GUK[AAAA]}$	YEAV774-777 to AAAA
pMT-Ed-GFP-$Dlg^{PDZ3\text{-}SH3\text{-}HOOK\text{-}GUK[AAN]}$	EEN768-770 to AAN
pMT-Ed-GFP-$Dlg^{PDZ3\text{-}SH3\text{-}HOOK\text{-}GUK[AAW]}$	EWW641-643 to AAW
pMT-Ed-GFP-$Scrib^{LRR+LAPSD}$	Fragment encompassing aa1-715

Table S3. List of genotypes used in Figures

Figure	Genotype
Fig. 1B	hsFLP[122]/+;+/UAS-Dlg[WT]::HA;act>y+>GAL4,UAS-his2av::mRFP/+
Fig. 1C	hsFLP[122]/+;+/UAS-Dlg[ASAKA]::HA;act>y+>GAL4,UAS-his2av::mRFP/+
Fig. 1D	hsFLP[122]/+;UAS-scrib RNAi 39073/UAS-Dlg[WT]::HA;act>y+>GAL4,UAS-his2av::mRFP/+
Fig. 1E	hsFLP[122]/+;UAS-scrib RNAi 39073/UAS-Dlg[ASAKA]::HA;act>y+>GAL4,UAS-his2av::mRFP/+
Fig. 1G	d174-GAL4,dlg[40.2]/+
Fig. 1H	d174-GAL4,dlg[40.2]/Y
Fig. 1I	d174-GAL4,dlg[40.2]/Y;+/UAS-Dlg[WT]::HA
Fig. 1J	d174-GAL4,dlg[40.2]/Y;+/UAS-Dlg[ASAKA]::HA
Fig. 1K	hsFLP[1],FRT19A tub-GAL80/dlg[m52],FRT19A;act-GAL4,UAS-GFP/+
Fig. 1L	hsFLP[1],FRT19A tub-GAL80/dlg[m52],FRT19A;act-GAL4,UAS-GFP/UAS-Dlg[WT]::HA
Fig. 1M	hsFLP[1],FRT19A tub-GAL80/dlg[m52],FRT19A;act-GAL4,UAS-GFP/UAS-Dlg[ASAKA]::HA
Fig. 2A	
Fig. 2C	hsFLP[122]/+;+/UAS-Lgl::GFP;act>y+>GAL4,UAS-his2av::mRFP/+
Fig. 2D	hsFLP[122]/+;UAS-dlg RNAi 39035/UAS-Lgl::GFP;act>y+>GAL4,UAS-his2av::mRFP/+
Fig. 2E	hsFLP[122]/+;+/UAS-Lgl[KAFA]::GFP;act>y+>GAL4,UAS-his2av::mRFP/+
Fig. 2F	hsFLP[122]/+;UAS-dlg RNAi 39035/UAS-Lgl[KAFA]::GFP;act>y+>GAL4,UAS-his2av::mRFP/+
Fig. 4A	TJ-GAL4/UAS-Dlg[WT]::HA
Fig. 4B	TJ-GAL4/UAS-Dlg[SH3-HOOK-GUK]::HA
Fig. 4C	TJ-GAL4/UAS-Dlg[SH3-HOOK]::HA
Fig. 4E	d174-GAL4,dlg[40.2]/+
Fig. 4F	d174-GAL4,dlg[40.2]/Y
Fig. 4G	d174-GAL4,dlg[40.2]/Y;+/UAS-Dlg[SH3-HOOK-GUK]::HA
Fig. 4H	d174-GAL4,dlg[40.2]/Y;+/UAS-Dlg[SH3-HOOK]::HA
Fig. 4I	hsFLP[122]/+;UAS-dlg RNAi 39035/+;act>y+>GAL4,UAS-his2av::mRFP/+
Fig. 4J	hsFLP[122]/+;UAS-dlg RNAi 39035/UAS-Dlg[SH3-HOOK-GUK]::HA;act>y+>GAL4,UAS-his2av::mRFP/+
Fig. 4K	hsFLP[122]/+;UAS-dlg RNAi 39035/UAS-Dlg[SH3-HOOK-GUK]::HA;act>y+>GAL4,UAS-his2av::mRFP/+
Fig. 4M	hsFLP[122]/+;UAS-dlg RNAi 39035/+;act>y+>GAL4,UAS-his2av::mRFP/+
Fig. 4N	hsFLP[122]/+;UAS-dlg RNAi 39035/UAS-Dlg[SH3-HOOK-GUK]::HA;act>y+>GAL4,UAS-his2av::mRFP/+
Fig. 4O	hsFLP[122]/+;UAS-dlg RNAi 39035/UAS-Dlg[SH3-HOOK-GUK]::HA;act>y+>GAL4,UAS-his2av::mRFP/+
Fig. 5A	hsFLP[1],FRT19A tub-GAL80/dlg[m52],FRT19A;act-GAL4,UAS-GFP/+
Fig. 5B	hsFLP[1],FRT19A tub-GAL80/dlg[m52],FRT19A;act-GAL4,UAS-GFP/UAS-Dlg[WT]::HA
Fig. 5C	hsFLP[1],FRT19A tub-GAL80/dlg[m52],FRT19A;act-GAL4,UAS-GFP/UAS-Dlg[ASAKA]::HA
Fig. 5E	hsFLP[1],FRT19A tub-GAL80/dlg[m52],FRT19A;act-GAL4,UAS-GFP/+
Fig. 5F	hsFLP[1],FRT19A tub-GAL80/dlg[m30],FRT19A;act-GAL4,UAS-GFP/+

Fig. 5G	hsFLP[1],FRT19A tub-GAL80/dlg[m52],FRT19A;act-GAL4,UAS-GFP/UAS-myr-Scrib::V5
Fig. 5H	hsFLP[1],FRT19A tub-GAL80/dlg[m52],FRT19A;act-GAL4,UAS-GFP/UAS-myr-Scrib::V5
Fig. S1A,C	TJ-GAL4/+
Fig. S1B,D	TJ-GAL4/+;+/UAS-Pp1-87B RNAi 32414
Fig. S1F	hsFLP[122]/+;UAS-dlg RNAi 39035/+;act>y+>GAL4,UAS-his2av::mRFP/+
Fig. S1G	hsFLP[122]/+;UAS-dlg RNAi 39035/+;act>y+>GAL4,UAS-his2av::mRFP/UAS-Pp1-87B::HA
Fig. S2B	hsFLP[122]/+;+/UAS-myr-Scrib::V5;act>y+>GAL4,UAS-his2av::mRFP/+
Fig. S2C	hsFLP[122]/+;+/UAS-myr-Scrib[TAAA/RAGA]::V5;act>y+>GAL4,UAS-his2av::mRFP/+
Fig. S2E	d174-GAL4/+;scrib[2]/+
Fig. S2F	d174-GAL4/+;scrib[2]/scrib[1]
Fig. S2G	d174-GAL4/+;scrib[2]/scrib[1];+/UAS-myr-Scrib::V5
Fig. S2H	d174-GAL4/+;scrib[2]/scrib[1];+/UAS-myr-Scrib[TAAA/RAGA]::V5
Fig. S2I	hsFLP/+;act-GAL4,UAS-GFP/UAS-myr-Scrib::V5;tub-GAL80,FRT82B/scrib[1],FRT82B
Fig. S2J	hsFLP/+;act-GAL4,UAS-GFP/UAS-myr-Scrib[TAAA/RAGA]::V5;tub-GAL80,FRT82B/scrib[1],FRT82B
Fig. S4A	hh-GAL4/+
Fig. S4B	+/UAS-dlg RNAi 39035;hh-GAL4/+
Fig. S4C	UAS-dlg RNAi 39035/UAS-Dlg[SH3-HOOK-GUK]::HA;hh-GAL4/+
Fig. S4D	UAS-dlg RNAi 39035/UAS-Dlg[SH3-HOOK]::HA;hh-GAL4/+
Fig. S6A	hsFLP[122]/+;UAS-scrib RNAi 39073/lgl[S5A]::GFP;act>y+>GAL4,UAS-his2av::mRFP/+
Fig. S6B	hsFLP[122]/+;UAS-dlg RNAi 39035/lgl[S5A]::GFP;act>y+>GAL4,UAS-his2av::mRFP/+

Biology Open • Supplementary information

Supplemental References

Bilder, D. and Perrimon, N. (2000). Localization of apical epithelial determinants by the basolateral PDZ protein Scribble. *Nature* **403**, 676–680.

Dong, W., Zhang, X., Liu, W., Chen, Y. jiun, Huang, J., Austin, E., Celotto, A. M., Jiang, W. Z., Palladino, M. J., Jiang, Y., et al. (2015). A conserved polybasic domain mediates plasma membrane targeting of Lgl and its regulation by hypoxia. *J. Cell Biol.* **211**, 273–286.

Garcia, J. D., Dewey, E. B. and Johnston, C. A. (2014). Dishevelled binds the Discs large "Hook" domain to activate GukHolder-dependent spindle positioning in Drosophila. *PLoS One* **9**, 1–17.

Johnston, C. A., Hirono, K., Prehoda, K. E. and Doe, C. Q. (2009). Identification of an Aurora-A/PinsLINKER/ Dlg Spindle Orientation Pathway using Induced Cell Polarity in S2 Cells. *Cell* **138**, 1150–1163.

Khoury, M. J. and Bilder, D. (2020). Distinct activities of Scrib module proteins organize epithelial polarity. *Proc. Natl. Acad. Sci.* **117**, 11531–11540.

Koh, Y. H., Popova, E., Thomas, U., Griffith, L. C. and Budnik, V. (1999). Regulation of DLG localization at synapses by CaMKII-dependent phosphorylation. *Cell* **98**, 353–363.

Lee, T. and Luo, L. (1999). Mosaic analysis with a repressible cell marker for studies of gene function in neuronal morphogenesis. *Neuron* **22**, 451–61.

Mendoza-Topaz, C., Urra, F., Barría, R., Albornoz, V., Ugalde, D., Thomas, U., Gundelfinger, E. D., Delgado, R., Kukuljan, M., Sanxaridis, P. D., et al. (2008). DLGS97/SAP97 is developmentally upregulated and is required for complex adult behaviors and synapse morphology and function. *J. Neurosci.* **28**, 304–314.

Moreira, S., Osswald, M., Ventura, G., Gonçalves, M., Sunkel, C. E. and Morais-de-Sá, E. (2019). PP1-Mediated Dephosphorylation of Lgl Controls Apical-basal Polarity. *Cell Rep.* **26**, 293–301.

Perrimon, N. (1988). The maternal effect of lethal(1)discs-large-1: A recessive oncogene of Drosophila melanogaster. *Dev. Biol.* **127**, 392–407.

Sharp, K. A., Khoury, M. J., Wirtz-Peitz, F. and Bilder, D. (2021). Evidence for a nuclear role for Drosophila Dlg as a regulator of the NURF complex. *Mol. Biol. Cell* **32**,.

Tanimoto, H., Itoh, S., Ten Dijke, P. and Tabata, T. (2000). Hedgehog creates a gradient of DPP activity in Drosophila wing imaginal discs. *Mol. Cell* **5**, 59–71.

Wirtz-Peitz, F., Nishimura, T. and Knoblich, J. A. (2008). Linking Cell Cycle to Asymmetric Division: Aurora-A Phosphorylates the Par Complex to Regulate Numb Localization. *Cell* **135**, 161–173.

Woods, D. F. and Bryant, P. J. (1989). Molecular cloning of the lethal(1)discs large-1 oncogene of Drosophila. *Dev. Biol.* **134**, 222–235.

Zeitler, J., Hsu, C. P., Dionne, H. and Bilder, D. (2004). Domains controlling cell polarity and proliferation in the Drosophila tumor suppressor scribble. *J. Cell Biol.* **167**, 1137–1146.

Biology Open • Supplementary information
